# Postpartum Contraception in Adolescents: Data From a Single Tertiary Clinic in Southeast of Turkey

**DOI:** 10.5539/gjhs.v7n2p80

**Published:** 2014-09-28

**Authors:** Mustafa Kaplanoglu, Dilek Kaplanoglu, Mustafa Gokhan Usman

**Affiliations:** 1 Adiyaman University School of Medicine, Department of Obstetrics and Gynecology, Adiyaman, Turkey; 2 Adiyaman University School of Medicine, Department of Familiy Physician, Adiyaman, Turkey

**Keywords:** adolescent, pregnancy, postpartum contraception

## Abstract

**Aim:**

We aimed to evaluate the postpartum contraception preferences of adolescent women in this study.

**Material and Method:**

This descriptive study was prepared after a retrospective analysis of file records of primigravida women who had given birth at the Adiyaman University School of Medicine Training and Research Hospital Department of Obstetric and Gynecology between January 2010 and June 2012. More than 12 months had passed after birth. The adolescents who were included in the study and the control group women were called by phone and invited to our clinic. A total of 506 adolescents and 1,046 control group women came to the clinic and were evaluated. The control group was formed of women between the age of 20-35 years who gave given birth in our clinic during the same period and were randomly selected. Postpartum obstetric history, contraception methods and data of these patients were recorded.

**Results:**

The mean age was 18.3±0.4 years and 28.2±4.9 years in the adolescent group and control group respectively. No contraception other than lactation amenorrhea was used by 256 women of the adolescent group (50.6%) and 345 women of the control group (33%). The most commonly used contraceptive method in both groups other than lactation amenorrhea was condoms (160 women (64%) and 230 women (32.8%) respectively). The annual contraceptive failure rate was 3.95% in the adolescent group and 1.72% in the control group. The highest failure rate was with lactation amenorrhea in both groups.

**Discussion:**

Adolescent women mostly use contraceptive methods with low reliability such as lactation amenorrhea and the calendar method in the postpartum period. Providing adequate contraceptive education is therefore important. On the other hand, starting such training starting in the early postnatal period will prevent recurring adolescent pregnancies with a short pregnancy interval.

## 1. Introduction

Improvements in birth control methods and access to relevant information in the last 20 years have contributed to the decrease of unintended pregnancies and consequent maternal and fetal complications in Turkey (Turkey Demographic and Health Survey, 2008). Contraception is especially significant for the adolescent age group and the postpartum period in all women. The lack of information on contraception knowledge as well as the ovulation return time due to postpartum lactation amenorrhea and the limited contraception options for this age group lead to an increased frequency of unintended pregnancies, especially in developing countries (Bozkurt et al., 2006; Clark et al., 2014; Chandra-Mouli et al., 2014). The short interval for these unintended pregnancies is especially associated with serious maternal and fetal morbidity and mortality in adolescents. However, the number of studies on adolescent women’s postpartum contraception preferences as related to these subjects is inadequate. The rate of adolescent pregnancies in developing countries is still very high compared with the rate in developed countries. This difference may be due to the lack of information on postpartum contraception knowledge in the adolescent age group. Contraceptive education for adolescent women may decrease the rate of postpartum unintended pregnancies. We aimed to evaluate the postpartum contraception preferences and their efficiency in adolescents in our study.

## 2. Materials and Methods

Women with full birth records and contact details among the primigravida patients who had given birth at the Adiyaman University School of Medicine Training and Research Hospital Department of Obstetrics and Gynecology between January 2010 and June 2012 were evaluated. Women who were still in adolescence during the study (10–19 years of age) were included in the study as the adolescent group. The women who met the inclusion criteria were called by phone and invited to our clinic for a follow-up. The inclusion criteria were the ability to reach the patient and fully obtain data, at least 12 months having passed since the patient gave birth, and a lack of surgical or medical problems that required discontinuing the contraceptive method used within the specified time. Of the adolescent women who were invited to our clinic, 506 patients came for the evaluation and were included in the study. The control group was formed by randomly selecting 1,046 patients within the age group of 20–35 years who had given birth in our clinic at the specified time and were invited to our clinic. The patients live in same area of Turkey. There was no medical and surgical problems in these patients. The demographic features of the groups are shown in Table 1.

**Table 1:** Demographic features of groups

		Adolescent	Control	P
Age (year)		18,3±0,4	28,2±4,9	< 0,05
Marital Status n(%)				0,501
	Married	495 (97,8)	1016 (97,1)	
	Unmarried	11 (2,2)	30(2,9)	
Educational level n(%)				0,332
	Low (≤ 8 yr)	421 (83,2)	890 (85,1)	
	High (> 8 yr)	85 (16,8)	156 (14,9)	
Urban or rural area n(%)				0,245
	Urban	189 (37,4)	424 (40,5)	
	Rural	317 (62,6)	622 (59,5)	
Gestational age at delivery (wk)		38,1±1,6	38±2,1	> 0,05
Breastfeeding period (month)		10±5,3	11,1±5,7	< 0,05
First examination (wk)		13,6±3,6	11,3±2,2	0,02

*Note.* n= number of patients, wk= week, yr= year.

Patient information acquisition started with the screening of hospital records and files in the first stage of evaluation. The age of the patient and the form of birth were recorded. In the second step, current information about contraception methods used in the postpartum period and the total duration of breastfeeding was obtained (by MK and DK) by asking the patient directly. The ratio of use and the success rate of each contraceptive method were evaluated. During this period, the hospital records regarding any new pregnancies were evaluated in patients who had become pregnant again.

In this study, an informed consent form was first read by the patients’ husbands or parents. After that, every question about study procedure was answered by a doctor (by MK or DK). Second, the form was signed by the patients, their husbands or parents, and the doctor. All patients’ records have been routinely entered into a hospital and government database. Study approval was obtained from the Adiyaman University School of Medicine Ethical committee

Statistical analyses were performed using the Statistical Package for the Social Sciences software version 13.0 (SPSS Inc., Chicago, Ill., USA). Comparisons between two groups possessing normally distributed variables were performed with the *independent samples t test*. Groups comprising categorical variables were compared with Pearson’s Chi-Square test. The level of statistical significance was defined as p<0.05.

## 3. Results

A total of 15,761 births occurred in our clinic during the specified time interval, and the adolescent birth ratio in the same period was 7.42%. The 506 adolescent patients who met the inclusion criteria and whose data we could access and 1,046 control group patients were included in the study. The mean pregnancy week at birth was 38.2±1.6 weeks in the adolescent age group and 38±2.1 weeks in the control group. No statistically significant difference in mean pregnancy week at birth was found between the groups (p>0.05). No statistically significant difference was found between the groups in terms of the type of birth either; normal vaginal birth rates were 88.7% and 90.4% for the adolescent and control groups, respectively (p>0.05). We did find a statistically significant difference between the groups in terms of the duration of breastfeeding (p<0.05). Breastfeeding duration in the adolescent age group (10±5.3 months) was shorter than in the control group (11.1±5.7 months) (Table 1).

A statistically significant difference was again found between the groups in terms of contraception preferences (p<0.05) (Table 2). This difference was particularly marked in the rate of patients who did not use any method other than lactation amenorrhea, which was 50.6% (256 patients) of the adolescent age group and 33% (345 patients) of the control group. The most commonly used method was condom use, with 64% (160 patients) of the adolescent age group and 32.8% (230 patients) of the control group choosing this method when patients who used a contraceptive method (without lactational amenorrea) were evaluated. The least commonly used methods were progesterone-containing pills, used by 4% (10 patients) of the adolescent group, and the calendar method, used by 11% (77 patients) of the control group (Table 3).

**Table 2:** Contraceptive use of groups

	Lactational Amenorrehae	Other methods
Adolescent group n(%)	256 (50,6)	250 (49,4)
Control group(%)	345(33)	701 (67)

*Note.* n= number of patients.

**Table 3:** Use of other methods in each group

	Progesterone containing pill	IUD	Calender method	Condom	Enjektabl Contasep	Total
Adolescent group n(%)	10 (4)	22(8,8)	36(14,4)	160(64)	22(8,8)	250
Control group n(%)	99(14,1)	131(18,7)	77(11)	230(32,8)	164(23,4)	701

*Note.* n= number of patients.

There were 20 unintended pregnancies in the adolescent group, and 18 in the control group were found within the specified time (the contraception failure rate was 3.95% and 1.72%, respectively). Sixty percent (12 patients) of the adolescent group had not used any contraceptive method except lactation amenorrhea, while 15% (three patients) were found to be pregnant despite using condoms and 25% (five patients) despite using the calendar method. Of the pregnancies in the control group, 55.5% (10 patients) had occurred during the use of lactation amenorrhea, 38.8% (7 patients) with the calendar method, and 5.55% (1 patient) during condom use. In addition, evaluation of the detection weeks of unwanted pregnancies showed a statistically significant difference between the groups, with 13.6±3.6 weeks and 11.3±2.2 weeks in the adolescent and control groups respectively.

## 4. Discussion

The adolescent age group is important to the health of the general public in many aspects (such as preterm labor, postpartum hemorrhage). Current studies have shown that about 82% of the pregnancies in this age group are not desired, and 31% end in abortion (Finer & Zolna, 2011; Kost, & Henshaw, 2012). Ongoing pregnancies are associated with serious maternal and fetal complications (Arkan et al., 2010). Contraception is therefore quite important for this age group. The main reasons for pregnancies in this age group are socio-cultural characteristics and adolescents not being sufficiently educated about contraception (Chandra-Mouli et al., 2014).

Postpartum contraception is generally required after the third month postpartum because the beginning of ovulation after delivery is not known (Kennedy & Visness, 1992). Although lactation amenorrhea is still the most widely used method of postpartum contraception, the fact that ovulation start time is unknown and the need for frequent and regular breastfeeding often cause this method to fail to provide adequate protection. The protection further decreases when the child starts taking in additional nutrients and the frequency and intensity of breastfeeding decreases (Kazi et al., 1995; Shaaban et al., 2013). When we compared the breastfeeding durations between the groups, the duration was shorter in adolescent women than in the control group (10±5.3 months and 11.1±5.7 months, respectively). This is a serious problem, especially for women who use lactation amenorrhea as a contraceptive method. The short breastfeeding duration can be explained by the inadequate knowledge of women in this age group about the potential benefits of breastfeeding and also stopping breastfeeding as a result of becoming pregnant in a short time (Kazi et al., 1995). A review has found a high protection rate in the first six months with regular breastfeeding (Kazi et al., 1995). However, the number of studies evaluating the postpartum contraceptive efficiency of lactation amenorrhea in adolescent women is not enough. Although the most preferred method of postpartum contraception was lactation amenorrhea in the specified time interval in our study, most unintended pregnancies in the adolescent age group occurred among the women using this method (60%). There were 12 unintended pregnancies in the first year with this method, and the contraceptive failure ratio was 2.37%. This rate was 2.02% in the control group. Other studies evaluating the efficiency of lactation amenorrhea without distinction of age have similarly reported that about 52% of women use this method for contraception in the postpartum period, and the mean annual pregnancy rate is 1.1% (Kazi et al., 1995; Sipsma, Bradley, & Chen, 2013). Another important issue is that these patients recognized their new pregnancies late because they were in the amenorrheic period. The mean outpatient presentation time of adolescent patients who became pregnant while using lactation amenorrhea as a contraception method was 13.6±3.6 weeks in our study. This time is generally too late for a dual screening test to be used for routine pregnancy screening and makes it impossible to use the test.

Depot medroxyprogesterone acetate (MPA) injection is a long-term contraceptive method known to be effective. It also increases milk, especially during lactation. It is associated with a reversible reduction in bone mineral density in the adolescent age group (Isley MM & Kaunitz AM, 2011). However, it can be used for longer than two years in patients who cannot use other methods (U.S. Food and Drug Administration, 2009). The most significant problems are weight gain and irregular bleeding. Metrorrhagia-type bleedings can lead to amenorrhea in the future (Cromer et al., 1996; O’Dell et al., 1998). The drug is preferred as there is no need to take medication regularly or provide additional training. There is a limited number of studies in the literature regarding the use of MPA as a long-acting contraceptive during the postpartum period in adolescents. Continuation of the method was observed 80% in the first six months and 42.9% in the twelfth month in these studies, and no unwanted pregnancy was found during the study. The patients fully complied with the method in the submitted study, and no unintended pregnancy was found, which is consistent with the literature (Howard, Wayman, & Strickland, 2013). The high continuation rate may be due to the bleeding’s not being serious enough to warrant discontinuation and the close monitoring and increased awareness provided by the health personnel.

The combined type of oral contraceptive pills are the most commonly used contraceptive method in the adolescent age group today after the condom. An annual failure rate up to 8% was found in adolescents. (French RS & Cowan FM, 2009). The use of contraceptive pills containing progesterone only is especially common in parous women and in the postpartum and lactation periods. Decreasing the amount of bleeding is a reason for this preference, especially in anemic patients (Pregnancy Risk Assessment Monitoring System (PRAMS), 2012; ACOG Committee on Practice Bullitens—Gyncology, 2001). Although the precise rate of use is not known, it has been reported as 0.4% in the 14–44 years age range in the U.S. (Hall, Trussell, & Schwarz, 2012). The annual failure rate of these pills is 0.27% (95% CI 0.06 to 1.19). Menstruation irregularities were found with their use, but the rate was not statistically significant (Grimes et al., 2010). The utilization rate in the adolescent group was 1.9% in our study, and no unintended pregnancy occurred. Using condoms is easy and is a cheap and efficient contraceptive method. It is the most commonly used contraceptive method in the adolescent age group (Martinez et al., 2011; Verhaeghe, 2012). However, a failure rate of up to 17% is reported with incorrect use (Williams & Fortenberry, 2011). The annual failure rate was 1.87% in our study. Condoms do have more advantages than other medical contraceptive methods. A spouse’s use of condoms can help women avoid daily pill stress and the use of different pharmacological agents (Kaplan et al., 2001). It is a method that can be used successfully for a long time after a short training period. Protection against sexually transmitted diseases is a secondary gain. Condom use as high as 64% was observed in our adolescent group. However, three unintended pregnancies occurred, which were probably due to incorrect use.

The calendar method and intrauterine devices (IUDs) are other contraception methods that can be used in the postpartum period. The ovulation time not being known with certainty is a significant problem for the calendar method. In addition, the compliance and motivation of the patient and the spouse are important for the calendar method. Increased motivation has been shown to increase the success rate (Fehring et al., 2013; Abma et al., 2010). It is necessary to know the cycle pattern and monitor ovulation findings to increase effectiveness. In our study, five unintended pregnancies were identified in the first year in patients who used this method (annual failure ratio 13.8%). The annual failure rate was 18.5% in a meta-analysis of eight studies (Kambic & Lamprecht, 1996). IUDs are used in postpartum period as an effective long-term contraceptive method that does not require prophylactic antibiotics (ACOG Committee Opinion No. 392, 2007). However, problems such as limited access to healthcare institutions and sociocultural restrictions, as well as possible complications such as pain and bleeding restrict their use (Allen et al., 2009). The IUD use rate was 8.8% in the adolescent group in our study. No pregnancy was observed during the period evaluated. The expulsion rate and unintended pregnancy rate were found as 15% and 10% in several studies in 24 months (Patchen & Berggren, 2011). The annual rate of IUD expulsion is generally accepted as 5–15% for application six weeks after birth (Goldstuck & Steyn, 2013). We had no patient whose IUD was expelled in our study and no unintended pregnancy in this group. This may be due to the difference in the observation durations between studies.

Adolescent pregnant women who have many complications are a major public health problem in every country. The gradual decrease in intercourse age in developing countries and the higher number of partners have been reported in various publications (Hanson, 1996). However, a decline has been found in adolescent pregnancy rates with intensive training programs on contraception. In addition, the potential complications of pregnancy and the resultant public costs have been reduced with these programs. However, adolescent marriages are still widespread in undeveloped and developing countries for socio-cultural reasons, so the adolescent pregnancy rate has not yet declined to the desired level (Tornello, Riskind, & Patterson, 2014).

Most adolescent postpartum patients use only the lactation amenorrhea method, which does not provide adequate contraception. Adolescent marriages are a major health problem, and insufficient knowledge about contraceptive methods leads to unintended adolescent pregnancies and complications (Mollen et al., 2012; Lemay et al., 2007). Reducing the number of adolescent marriages by providing basic education and ensuring that married couples receive adequate contraceptive education are important for public health. Such a reduction and education will play a significant role in the prevention of unintended pregnancies that may occur in the postpartum period. In this study, the population was limited. Therefore, studies with a larger number of patients are required to evaluate the data clearly.
